# Plasmonic Au–Cu nanostructures: Synthesis and applications

**DOI:** 10.3389/fchem.2023.1153936

**Published:** 2023-03-08

**Authors:** Xiaohu Mi, Huan Chen, Jinping Li, Haifa Qiao

**Affiliations:** ^1^ Shaanxi Collaborative Innovation Center of TCM Technologies and Devices, Shaanxi University of Chinese Medicine, Xixian New Area, China; ^2^ School of Physics and Information Technology, Shaanxi Normal University, Xi’an, China; ^3^ College of Acupuncture and Tuina, Shaanxi University of Chinese Medicine, Xixian New Area, China

**Keywords:** Au-Cu nanostructures, surface plamon resonance, catalysis, plasmon-enhanced spectroscopy, photothermal conversion

## Abstract

Plasmonic Au–Cu nanostructures composed of Au and Cu metals, have demonstrated advantages over their monolithic counterparts, which have recently attracted considerable attention. Au–Cu nanostructures are currently used in various research fields, including catalysis, light harvesting, optoelectronics, and biotechnologies. Herein, recent developments in Au–Cu nanostructures are summarized. The development of three types of Au–Cu nanostructures is reviewed, including alloys, core-shell structures, and Janus structures. Afterwards, we discuss the peculiar plasmonic properties of Au–Cu nanostructures as well as their potential applications. The excellent properties of Au–Cu nanostructures enable applications in catalysis, plasmon-enhanced spectroscopy, photothermal conversion and therapy. Lastly, we present our thoughts on the current status and future prospects of the Au–Cu nanostructures research field. This review is intended to contribute to the development of fabrication strategies and applications relating to Au–Cu nanostructures.

## 1 Introduction

Because of the synergetic interaction between the two different types of metals, bimetallic nanostructures exhibit deeply interesting optical and electric properties, which have wide applications in plasmonic and catalytic fields (Gilroy et al., 2016; Cortes et al., 2020; Skrabalak, 2021). It has been possible to prepare a variety of bimetallic nanostructures by using several different methods (Feng et al., 2012; Lee et al., 2014; Zheng et al., 2020; Zhou L. et al., 2021; Zhou M. et al., 2021; Feng et al., 2021; Jiang et al., 2021; Skrabalak, 2021; Yang et al., 2021; Lee et al., 2022). Cu and Au nanostructures are excellent materials for applications in catalysis and plasmonic (Zhang et al., 2019; Mi et al., 2021a; Xin et al., 2021; Zhang B. et al., 2022; Lin et al., 2022). Cu nanostructures, which are valuable plasmonic materials in the visible to near-infrared region, have contributed to the development of applications in photonics, sensing, heterogeneous catalysts and imaging (Gawande et al., 2016; Lee D. W. et al., 2021; Medvedeva et al., 2021). However, the Cu nanostructure has a strong tendency to oxidize upon exposure to air (Fan et al., 2021). For example, Au nanostructures show tunable optical properties by changing size and shape, which has stable chemical properties and strong photothermal and electromagnetic field enhancements (Hao et al., 2007; Zheng et al., 2021; Zare et al., 2022). Moreover, Au nanostructures can be applied in biosensing, drug delivery, and catalysis. The Au–Cu nanostructure is a novel bimetallic system that has potential applications in photothermal therapy, multimodal imaging, and heterogeneous catalysis fields by combining the advantages of Cu and Au nanostructures with tunable metallic composition and shape (Tong et al., 2019; Abbasi et al., 2020; Li et al., 2021; Cao et al., 2022).

This review aims to present and discuss recent advances in colloidal synthesis of controlled-shape Au–Cu bimetallic nanostructures and their emerging applications in photothermal therapy, catalysis, and plasmon-enhanced spectroscopy, among others. We will start with the introduction of Au–Cu nanostructures of various types. The unique plasmonic properties of the Au–Cu nanostructures can then be discussed. The three different kinds and the corresponding applications of the Au–Cu nanostructures will be presented in detail. Finally, our view of the current state of perspective and development of this research field will be presented.

## 2 Synthesis of Au–Cu nanostructures

In the past few decades, Au–Cu nanostructures have witnessed obvious advances in the colloidal synthesis of well-controlled sizes, shapes, structures, and compositions. In terms of atomic ordering, Au–Cu nanostructures can be divided into three major types: alloy, core-shell and Janus nanostructures (Ferrando et al., 2008; Wang and Li, 2011). Similar to other bimetallic nanostructures, the crystal structures, morphology and element distributions of the Au–Cu nanostructures are determined by the reaction pathways, which depend on the reaction conditions and synthetic method (Liu and Liu, 2012; Yu et al., 2014).

### 2.1 Au–Cu alloy nanostructures

**FIGURE 1:**
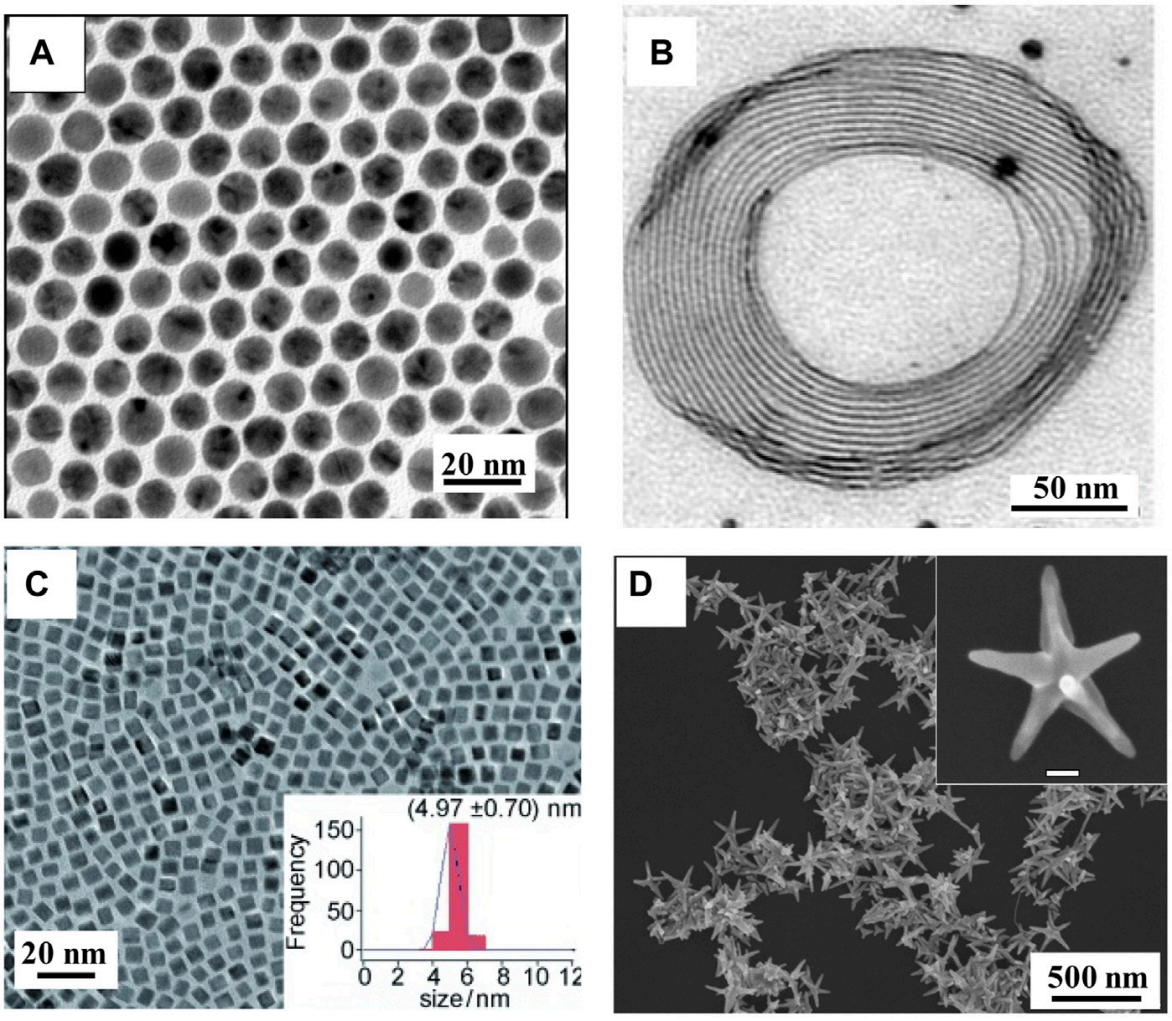
**(A)** TEM images of Au–Cu alloy nanospheres (Motl et al., 2010). **(B)** TEM images of Au–Cu alloy nanowires (Mendoza-Cruz et al., 2016). **(C)** TEM images of Au–Cu alloy nanocubes (Liu and Walker, 2010). **(D)** SEM image of Au–Cu alloy nanopentacles (He et al., 2014).

Because Au has similar characteristics to Cu (valence, atomic radii and crystal structure), the Au–Cu alloy can be easily prepared (Ferrando et al., 2008). The melting point of Au–Cu alloy nanostructures is lower than that of their constituent elements, and Au–Cu alloy melts at a specific temperature (Okamoto et al., 1987; Thota et al., 2018). Schaak and coworkers reported Au–Cu alloy nanospheres using a co-reduction method (Motl et al., 2010). During the preparation process, 1-octadecene, oleic acid, and oleylamine were chosen as the surfactants and reducing agents. A series of Au–Cu alloy nanospheres with different Au and Cu ratios were obtained by adjusting the concentration of the Cu precursor. As shown in Figure 1A, the uniform size distribution (8 nm) of alloy nanospheres is shown, indicating a disordered crystal structure. Au–Cu alloy nanowires have also been synthesized (Mendoza-Cruz et al., 2016). Alkylamine chains of ODA and glucose acted as surfactants and reducing agents, which helped the growth of alloy nanowires (Bazán-Díaz et al., 2018; Chatterjee et al., 2018). A coiled mode of Au–Cu alloy nanowires was self-assembled as individual nanowires or in a parallel-ordering manner as a set of nanowires. As shown in Figure 1B, the Au–Cu alloy nanowires are ultrathin, with diameters less than 10 nm and variable lengths of a few microns, presenting twin boundaries and an elevated density of stacked faults. The co-reduction method has been extended to obtain some complex geometrical nanostructures, such as Au–Cu alloy nanocubes (Figure 1C) (Liu and Walker, 2010). DDT also plays a critical role in controlling the morphology of Au–Cu alloy nanocubes. In addition, Xu and others reported the synthesis of Au–Cu alloy nanopentacles by combining two strategies (co-reduction and seed-mediated) in the aqueous phase route, with sizes that can be controlled in the 45 nm–200 nm range (Figure 1D) (He et al., 2014).

### 2.2 Au–Cu core-shell nanostructures

**FIGURE 2:**
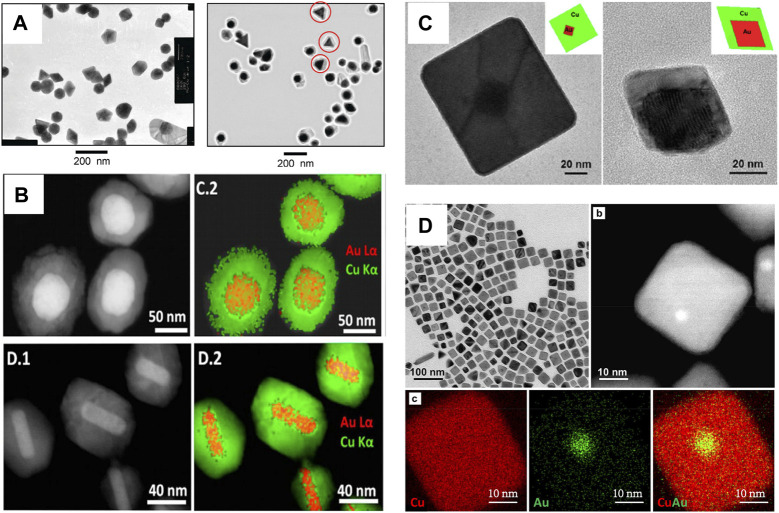
**(A)** TEM images of Au nanostructures and Au–Cu core-shell nanostructures (Tsuji et al., 2010). **(B)** HAADF-STEM images and STEM-EDS elemental maps of Au–Cu core-shell nanostructures (Alvarez-Paneque et al., 2012). **(C)** TEM images of the Au–Cu core−shell nanocubes and octahedra with Au octahedra nanocrystals as seeds (Kim et al., 2014). **(D)** TEM images and EDX mapping of Au–Cu core–shell nanocubes​(Lyu et al., 2019).

Bimetallic core–shell nanostructures offer numerous benefits; for example, the optical property is easily tuned by varying the morphology, shape, size, and composition of the core, as well as the thickness, shape, and composition of the shell material. Thus, the plasmonic property of the bimetallic core-shell nanostructure was easily regulated (Zhang Y.-J. et al., 2021; Wan et al., 2022). To date, although many attempts have been made to generate Au–Cu core–shell nanostructures by epitaxial growth with lattice mismatch, limited success has been achieved. For example, Tsuji and coworkers reported Au–Cu core–shell nanocrystals (Tsuji et al., 2010). The study showed that the Cu shell can epitaxially grow on the surface of Au nanocrystals, although a large lattice mismatch existed between Au and Cu for the first time (Figure 2A) (Wang et al., 2015). However, the Cu shell thickness and shape of the Au–Cu core–shell nanocrystal were not exactly regulated. Luis and collaborators reported the formation of Au–Cu core–shell nanostructures with uniform and various morphologies using Au nanostructures as templates (Alvarez-Paneque et al., 2012). The method is based on the reduction of Cu^2+^ in aqueous solution by hydrazine at 60°C. In addition, the size of the resulting Au–Cu core-shell nanostructure was tuned by either the size of the Au core or the ratio between the Cu and Au molarities (Figure 2B). Au–Cu core-shell nanostructures (nanocubes and nanooctahedra) with tunable sizes were synthesized in water by using Au nanooctahedra (Figure 2C) (Kim et al., 2014). The synthetic conditions were very simple, and nanoparticle growth was complete in 45–90 min. In their study, HDA not only increased the pH of the solution but also acted as a coordination ligand for Cu ions, facilitating controlled Cu shell growth. In addition, Xia’s group reported Au–Cu core-shell nanocubes, which exhibit a size smaller than 30 nm (Figure 2D) (Lyu et al., 2019).

### 2.3 Au–Cu janus nanostructures

**FIGURE 3:**
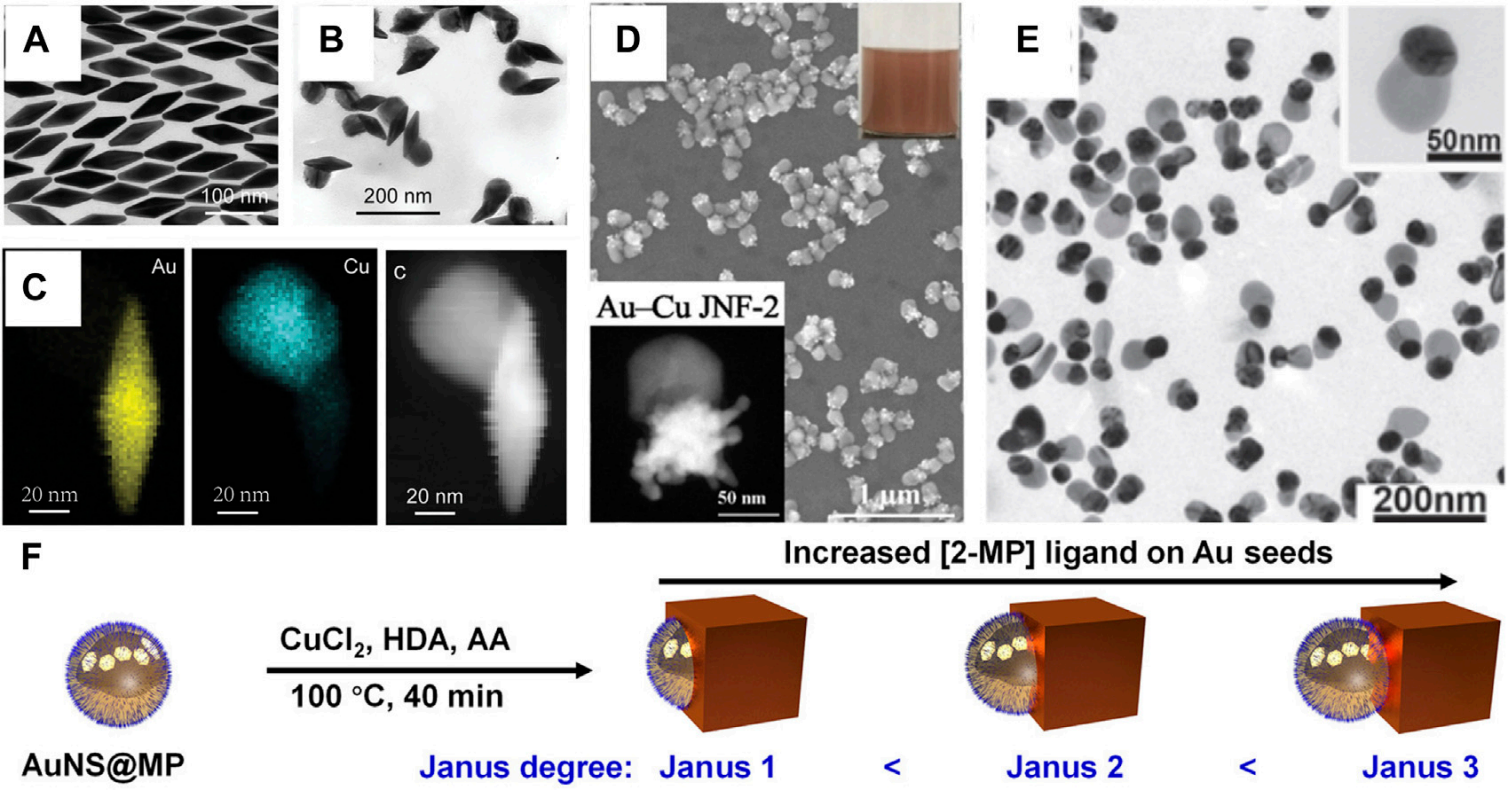
**(A–C)** Characterization of Au nanobipyramids and Au nanobipyramids–Cu nanostructures (Jia et al., 2021). **(A)** TEM image of Au nanobipyramids. **(B)** TEM image of Au nanobipyramids–Cu nanostructures. **(C)** Elemental maps and HAADF-STEM image of the Au NBP–Cu heterostructure. **(D)** HAADF-STEM image and SEM image of Au–Cu Janus nanojellyfish (Mi et al., 2022). **(E)** TEM images of Au–Cu heterodimers (Zhu et al., 2020). **(F)** Schematic illustrating the synthesis of Au–Cu Janus nanostructures (Fan et al., 2022).

The concept of the “Janus structure” was proposed by Pierre-Gilles de Gennes in 1991 (Gennes, 1992). Janus nanostructures not only reduce administered dosages but also combine differential functionalization (Zhang X. et al., 2021; Qiu et al., 2022). In all these protocols, the challenge for successful fabrication of bimetallic Janus nanostructures is how to create asymmetric distributions, where two metals are side-by-side in one particle, while avoiding the formation of different types of structures (Duan et al., 2021; Peng et al., 2021). It is very important to choose an appropriate capping agent in the shape-controlled synthesis of Au–Cu Janus nanostructures. The alkylamines and DNA can selectively bind to Cu, which helps Cu growth on the surface of the Au nanostructure (Liu and Fichthorn, 2017; Kim et al., 2021). For example, Zhang and coworkers proposed a general seed-mediated growth method for the synthesis of Au–Cu Janus nanostructures by HAD and CTAB as surfactants (Jia et al., 2021). The protocol can be trivially extended to various shapes of Au nanostructures as cores, such as Au nanobipyramids, suggesting the generality of the site-selective overgrowth method (Figures 3A–C). Similarly, our group designed a new jellyfish-like Au–Cu Janus nanostructure (Figure 3D) (Mi et al., 2022). A twin defect and stacking fault were found to exist at the twinned Au nanotips, which led to Cu atoms being deposited on the twinned nanotip. In addition, Deng’s group developed width-adjustable Au–Cu Janus nanostructures by fish sperm DNA (Figure 3E) (Zhu et al., 2020). The strategy relied on the non-specific surface adsorption of fish sperm DNA onto an Au nanoparticle to control heterogeneous Cu nucleation, which had low-cost and natural advantages. Such a process provided a chance to regulate the contact area between the Au nanoparticle and Cu nanodomains in the bimetallic nanostructure. Recently, the interfacial energy between Au nanostructures and Cu nanodomains was continuously regulated by strong thiol ligands, which induced a transition from Au–Cu core-shell nanostructures to Janus nanostructures (Fan et al., 2022). According to a series of effective ligand controls, Au–Cu Janus nanostructures were successfully prepared using different shapes of Au nanostructures as seeds. Crucially, the Janus degree of Au–Cu Janus nanostructures can be readily tuned by changing the molecular structure and the concentration of the thiol ligands (Figure 3F).

## 3 Optical properties

**FIGURE 4:**
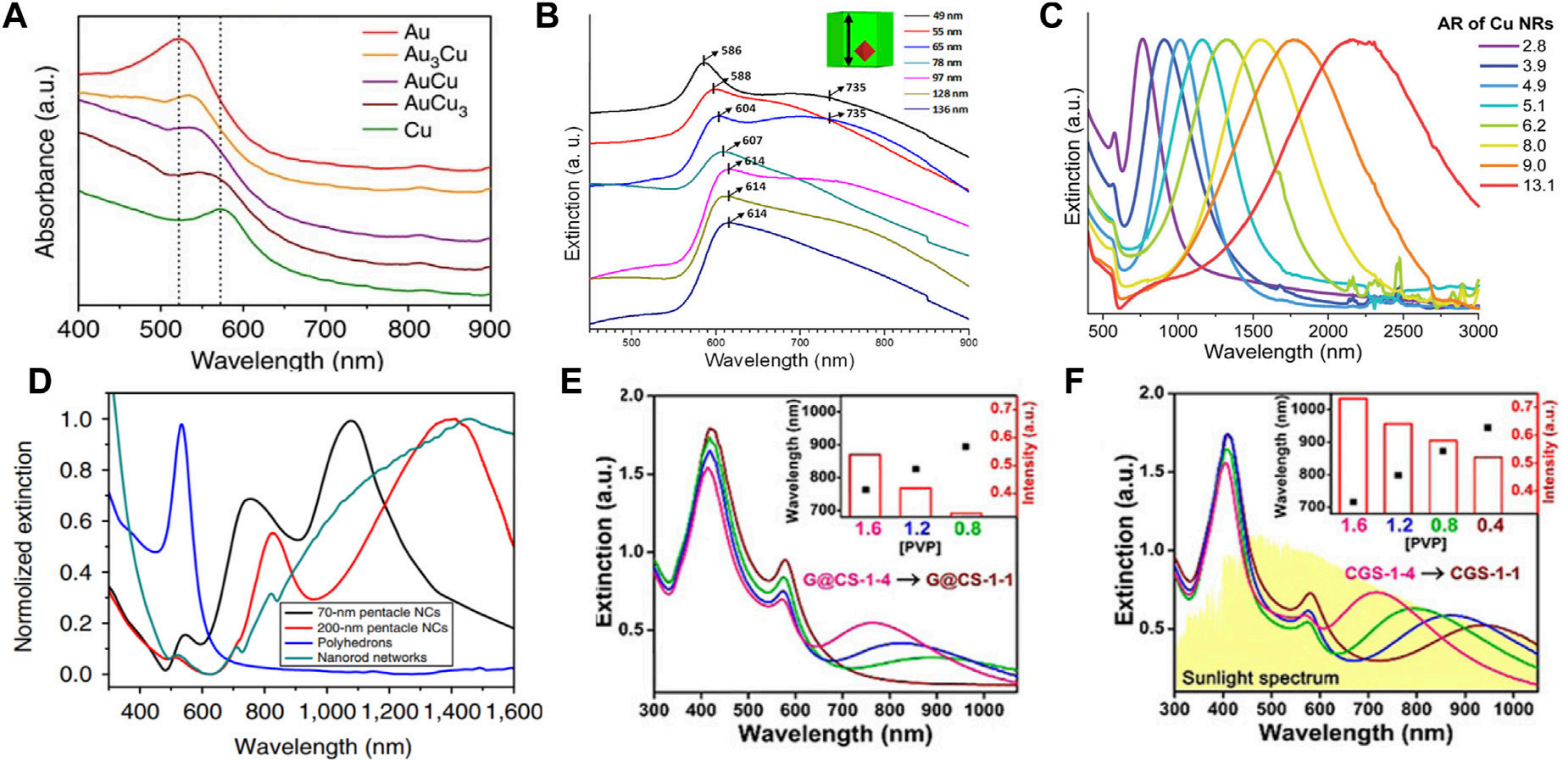
**(A)** Extinction spectra of Au nanospheres, Au–Cu alloy nanospheres and Cu nanospheres. Dotted lines indicate the LSPR peak of Au (∼520 nm) and Cu (∼570 nm) nanostructures (Kim et al., 2014). **(B)** Extinction spectra of Au–Cu core-shell cubes (Hsia et al., 2016). **(C)** Extinction spectra of Au–Cu NRs with different aspect ratios (Jeong et al., 2020). **(D)** Extinction spectra of Au–Cu alloy nanopentacles with different sizes (He et al., 2014). **(E)** and **(F)** Extinction spectra of Au–Cu–Ag nanostructures (G@CS-1 and CGS-1) with different concentrations of PVP(Lin et al., 2017).

Electrons in the conduction band of metallic nanostructures collectively oscillate under radiation from an external optical field. When the oscillation frequency of metallic nanostructures matches that of the light source, localized surface plasmon resonance (LSPR) occurs (Barnes et al., 2003; Ozbay, 2006). The frequency of LSPR is relevant to the morphology, size and composition of the metallic nanostructure and to the dielectric constant of the medium (Klar et al., 1998). For Au–Cu nanostructures, the wavelength and line shape of LSPR depends not only on their morphology and size but also on the Au and Cu elemental distribution (Mi et al., 2021a). Typically, this leads to a broadening of the LSPR and a well-defined redshift when the Cu content of the Au–Cu alloy nanosphere increases, as shown in Figure 4A. For example, the Au–Cu alloy nanospheres showed a single LSPR peak in the visible region. A clear redshift of the LSPR peak from 523 nm to 545 nm was observed when the Cu content was increased from 0% to 48% (Kim et al., 2014). A similar result was reported by Nicula and collaborators (Lungulescu et al., 2021). The LSPR absorption bands of Au–Cu core-shell nanocubes have been precisely controlled from 586 nm to 614 nm with changing edge lengths (Hsia et al., 2016). Compared with sphere counterparts, anisotropic Au–Cu nanostructures not only provide abundant LSPR modes but also focus more light to the nanogap and tip (Kneipp et al., 1997). For example, Au–Cu nanorods (NRs) displayed two modes of LSPR, which corresponded to the electron oscillations perpendicular and along the NR (Liebsch, 1993; Zheng et al., 2021). Hence, the longitudinal LSPR mode of Au–Cu NRs can be continuously shifted from the visible to the near-infrared (NIR) region by changing the aspect ratio of Au–Cu NRs (Ye et al., 2013; Luo et al., 2016; Mi et al., 2021b). For instance, the aspect ratio of the Au–Cu core-shell NR was readily tuned from 2.8 to 13.1 by varying the molar ratio between the Au NR and the Cu precursor, resulting in a wide range of LSPR wavelengths from 762 nm to 2201 nm (Figure 4C) (Jeong et al., 2020). The plasmonic nanostar is composed of several protruding nanotips and a central core, which usually show multiple LSPR modes corresponding to the tips and core−tip interactions. For example, 70 nm Au–Cu alloy nanopentacles showed three LSPR peaks (Figure 4D) (He et al., 2014). Two peaks of higher order modes were observed at 550 nm and 740 nm, while a major peak of dipolar mode was found at 1100 nm. Similarly, the LSPR of the 200 nm Au–Cu alloy nanopentacles showed three different peaks at 530 nm, 810 nm, and 1400 nm (red curve in Figure 4D). Moreover, Au–Cu nanostructures have been used as templates to design new multimetallic nanostructures (Chen et al., 2017; Shan et al., 2019; Dai et al., 2021). Au–Cu–Ag nanostructures had broadband optical absorption, which can cover the solar spectrum from the visible to infrared wavelength region by designing the configuration (Figures 4E,F(Lin et al., 2017). It has been found that the local electromagnetics of these nanostructures are precisely tuned in Au–Cu–Ag nanostructures.

To investigate the relation between the origin of the LSPR modes of Au–Cu nanostructures and their morphological features, single-particle spectroscopy was used to characterize individual Au–Cu nanostructures (Slaughter et al., 2011; Hartland and Shang Lo, 2013; Wang C. et al., 2021). The correlation between the number of arms of Au–Cu nanostars and scattering properties has been studied (Velazquez-Salazar et al., 2019). As the number of arms increases from one to two and three, multiple plasmonic bands appear and dominate the spectrum in the visible and near-IR regions, in agreement with previous reports on Au nanostars using plasmon hybridization theory (Prodan et al., 2003; Hao et al., 2007). In addition, scattering spectra of single Au–Cu alloy NRs were observed. It can be demonstrated that asymmetry and minor structural defects in Au–Cu alloy NRs induce multiple scattering peaks in a single Au–Cu alloy NR (Thota et al., 2015).

## 4 Applications

Due to the advantages of Au–Cu plasmonic nanoparticles with tunable composition and spatial distribution, more plasmonic modes of Au–Cu plasmonic nanoparticles can be generated (Li et al., 2010; Toscano et al., 2015; Zhuo et al., 2019). A wide range of applications have been reported in the fields of plasmon-enhanced electrocatalysis, surface-enhanced spectroscopy, phototherapy, and photocatalysis (Chen et al., 2019; Mi et al., 2019; Zhang B. et al., 2022; Zhang C. et al., 2022; Chen et al., 2022). In the following sections, different applications of Au–Cu nanostructures are presented and discussed in detail.

### 4.1 CO_2_ reduction

**FIGURE 5:**
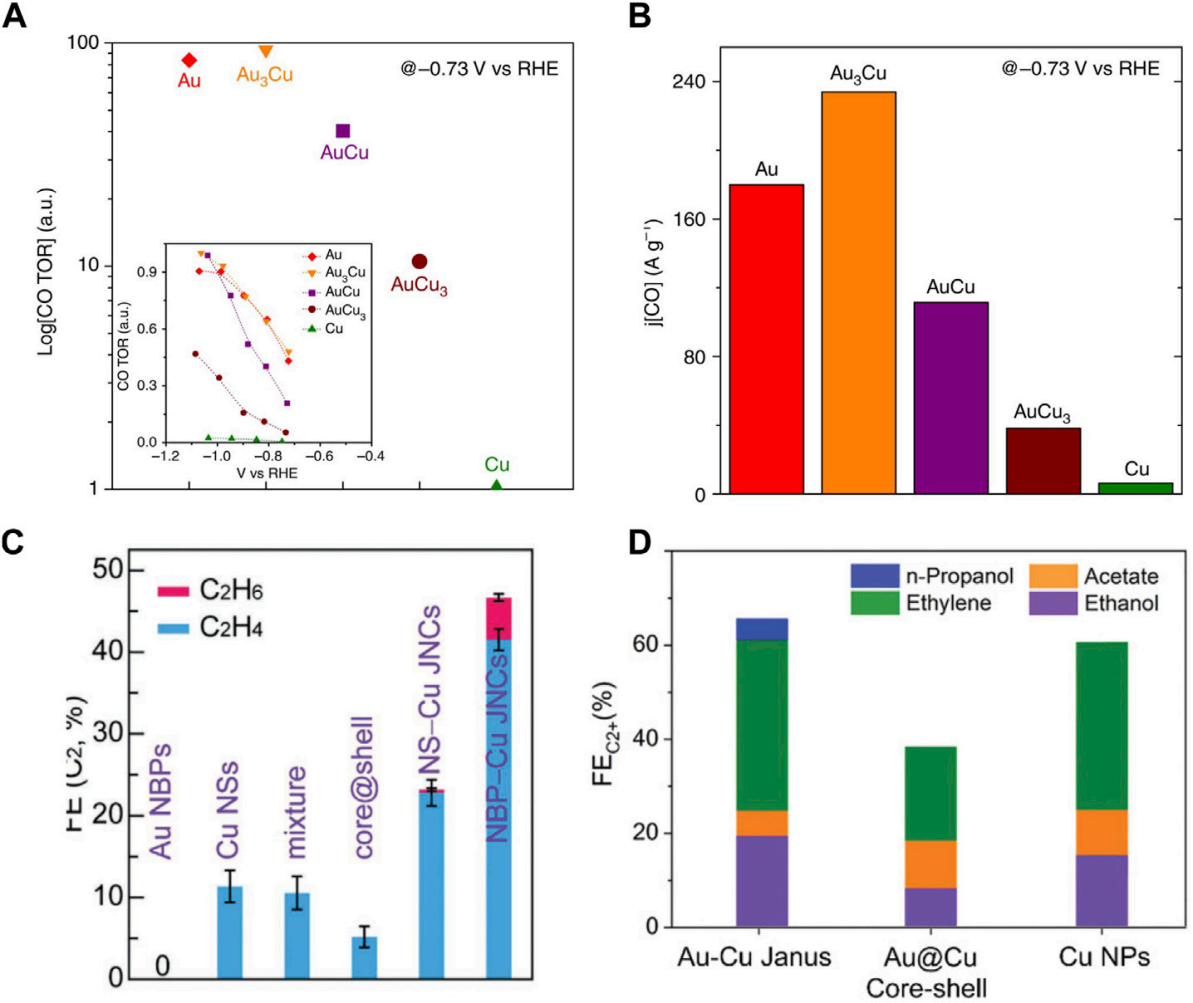
**(A,B)** Relative turnover rates and mass activity for CO of the Au–Cu alloy nanostructures (Kim et al., 2014). **(C)** Plot of faradaic efficiency for C_2_ products for Au bipyramids, Cu nanospheres, Au–Cu core-shell nanospheres, and Au nanosphere–Cu heterodimers (Jia et al., 2021). **(D)** Plot of faradaic efficiency for C_2_ products for Au–Cu Janus, Au–Cu core-shell and Cu nanoparticles (Zheng et al., 2022).

Electrochemical conversion of CO_2_ into feedstock and value-added fuels shows a convenient solution to energy demand and climate change (Nitopi et al., 2019). For this conversion, designing new catalysts with the capability to reduce CO_2_ into more valuable products is one of the challenges (Birdja et al., 2019; Wang et al., 2022). Although Cu nanoparticles have shown great helpe for CO_2_ reduction, poor selectivity persists (Lee S. H. et al., 2021). To solve this problem, the Au–Cu nanostructure has been exploited as a catalyst (Zhou et al., 2018; Ni et al., 2022). For example, alloying Au with Cu not only stabilizes Cu but also reduces the overpotential required for CO_2_ reduction. In addition to the excess potential, the selectivity of the catalysts during CO_2_ reduction must be considered since multiple couplings between protons and electrons lead to many possible reaction products (Bagchi et al., 2022). Yang’s group showed that Au–Cu alloy nanospheres are a highly potential catalyst for CO_2_ reduction (Kim et al., 2014). Catalytic activity and selectivity have been tuned by varying the composition of the Au–Cu alloy nanostructures. The Faraday efficiencies for hydrogen, ethylene, and methane increased with increasing Au content in alloy nanostructures, but the opposite results were obtained for carbon monoxide. As shown in Figure 5A, turnover rates of 93.1, 83.7, and 40.4 times for CO were obtained by Au_3_Cu, AuCu, and AuCu_3_ alloy nanospheres compared to Cu nanospheres. Au_3_Cu nanostructures also showed the best mass activity for CO (Figure 5B).

Additionally, due to tandem electrocatalysis, the Au–Cu Janus nanostructure provides an efficient conversion to the C_2_ product pathway (Jia et al., 2021). The symmetry importance was also shown by comparing different types of Au–Cu nanostructures, where the Janus nanostructure exhibited the best selectivity toward C_2_ products owing to the obvious boundaries between the Au and Cu nanodomains of the nanostructure. As shown in Figure 5C, the highest C_2_ product FEs of the Au nanobipyramide–Cu and Au nanosphere–Cu Janus nanostructures were 46.4% and 25.2%, which displayed 4.1-fold and 2.2-fold compared with that of the Cu nanosphere counterpart, respectively, showing the synergistic effect of Au and Cu in the Janus nanostructure. Furthermore, Huang’s group reported that the Au–Cu Janus nanostructure catalyst exhibited remarkable selectivity toward C_2_ product formation (Figure 5D) (Zheng et al., 2022).

### 4.2 Photothermal applications

**FIGURE 6:**
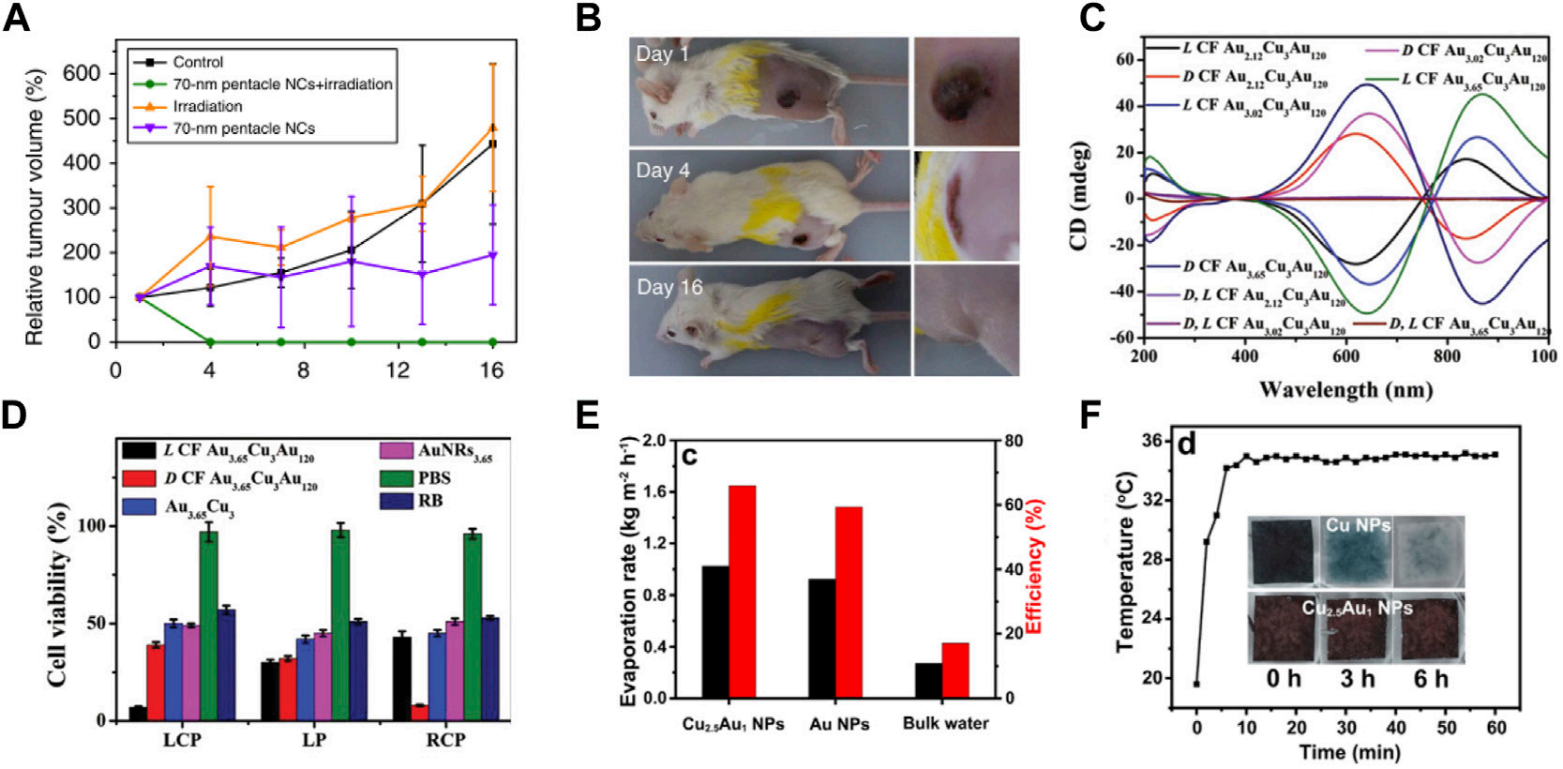
**(A,B)** Characterization of mouse tumor growth after treatment (He et al., 2014). **(A)** Tumor growth curves of different groups of mice after treatment. **(B)** Photographs of tumor-bearing mice at 1, 4 and 16 days after Au–Cu nanoparticle injection and irradiation in the 808 nm laser group. **(C,D)** CD and photothermal therapy properties of chiral Au–Cu–Au HNRs (Wang J. et al., 2020). **(C)** CD spectrum for different AuCuAu HNRs. **(D)** HeLa cell viability analysis after 808 nm left-circularly polarized, linear polarized and right-circularly polarized light irradiation of HeLa tumor-bearing mice after injection with L CF Au_3.65_–Cu_3_–Au_120_ HNRs. **(E–F)** Characterization of photothermal conversion properties and stability of Cu_2.5_–Au_1_ core-shell nanoparticles (Wang Y. et al., 2021). **(E)** The rates of solar evaporation and solar steam efficiency of Cu_2.5_–Au_1_ core-shell nanoparticles, Au nanoparticles, and bulk water under 1 sun illumination. **(F)** Time-dependent surface temperature curves of Cu_2.5_−Au_1_ nanoparticles placed on a saturated CO_2_ aqueous solution and illuminated by 1 sun. Insets in **(F)** show the photographs of Cu_2.5_−Au_1_ nanoparticles and Cu nanoparticles placed on a saturated CO_2_ solution with time.

For Au–Cu nanostructures, their LSPR peaks can also be tuned to the NIR region, making them good candidates for biomedical applications, such as bioimaging, photothermal therapy, and controlled drug release. As a new field, photothermal conversion and therapy are attracting extensive attention by using plasmonic nanostructures under NIR laser illumination (Zhu et al., 2021). Recently, Au–Cu systems have been explored for photothermal therapy. For example, mouse tumor cells were injected with Au–Cu nanopentacles. Then, mouse tumor cells were irradiated with an NIR laser. The tumor volume in the mice was also significantly reduced compared to tumor samples injected with Au–Cu nanoparticles only or irradiated with an 808 nm laser only. Under 808 nm laser irradiation, robust photothermal therapy efficiency was obtained by 70 nm Au–Cu nanopentacles. The relative tumor volume of mice after treatment along with feeding time is shown in Figure 6A. As seen in the purple and red segmentation lines, tumors from the groups injected with Au–Cu nanoparticles only and irradiated with 808 nm laser only were enlarged in volume and showed some fluctuations. The group of mice that received both Au–Cu nanopentacle irradiation and injection were tumor-free 4 days after treatment (Figure 6B). This work demonstrates that Au–Cu alloy nanostructures have potential applications in tumor diagnostics and therapeutics (He et al., 2014). In addition, the Cu–Au nanotripod also exhibited a well-defined prominent photothermal effect (Nanda et al., 2019). The cell viability results illustrated that Cu−Au nanotripods were minimally toxic to the cells. Au–Cu nanostructures also provide a chirality-dependent method for highly efficient phototherapy (Wang J. et al., 2020). The Au–Cu–Au heteronanorod (HNR) was synthesized by virtue of the dipeptide as ligands, which displayed strong circular dichroism (CD) in the range of 400–1000 nm (Figure 6C). The potential for photothermal and photodynamic therapy of chiral Au–Cu–Au HNRs was further investigated in HeLa cells by using confocal microscopy signals and CCK-8 assays. The Au–Cu–Au HNRs show the highest rate of cell toxicity (Figure 6D).

Solar steam generation is emerging as a promising technology, for its potential in harvesting solar energy for various applications such as desalination and sterilization (Hu et al., 2017; Xu et al., 2017). The studies has reported a variety of artificial structures that are designed and fabricated to improve energy conversion efficiencies by enhancing solar absorption, heat localization, water supply, and vapor transportation (Zhu et al., 2017). Recently, Cu−Au core-shell nanostructures showed strong broadband plasmonic absorption, which was used for solar steam generation (Wang Y. et al., 2021). The evaporation rate of water and photothermal efficiency of different plasmonic absorbers are shown in Figure 6E. The evaporation rates of the Cu_2.5_−Au_1_ nanoparticles and Au nanoparticles were 0.92 kg m^−2^ h^−1^ and 1.02 kg m^−2^ h^−1^ under 1 Sun irradiation for 5 h, respectively. Furthermore, a very high conversion efficiency of 66% was achieved by the Cu_2.5_−Au_1_ nanoparticles under 1 Sun irradiation compared to the Au nanoparticle absorber and bulk water. Next, the durability of Cu_2.5_−Au_1_ nanoparticles in a saturated CO_2_ solution was tested. Under 1 Sun irradiation, it can be observed that the color of Cu nanoparticles changed from black to colorless after 6 h (insets in Figure 6F). The reason is that Cu is easily oxidized. However, under the same test conditions, Cu_2.5_−Au_1_ nanoparticles remained black in color. Next, the temperature changes of the surface were tracked during the evaporation of saturated CO_2_ solutions at 1 solar. The temperature of the Cu_2.5_−Au_1_ nanoparticles remained constant under continuous illumination, implying excellent stability of the Cu_2.5_–Au_1_ nanoparticles (Figure 6F).

### 4.3 Plasmon-enhanced spectroscopies

**FIGURE 7:**
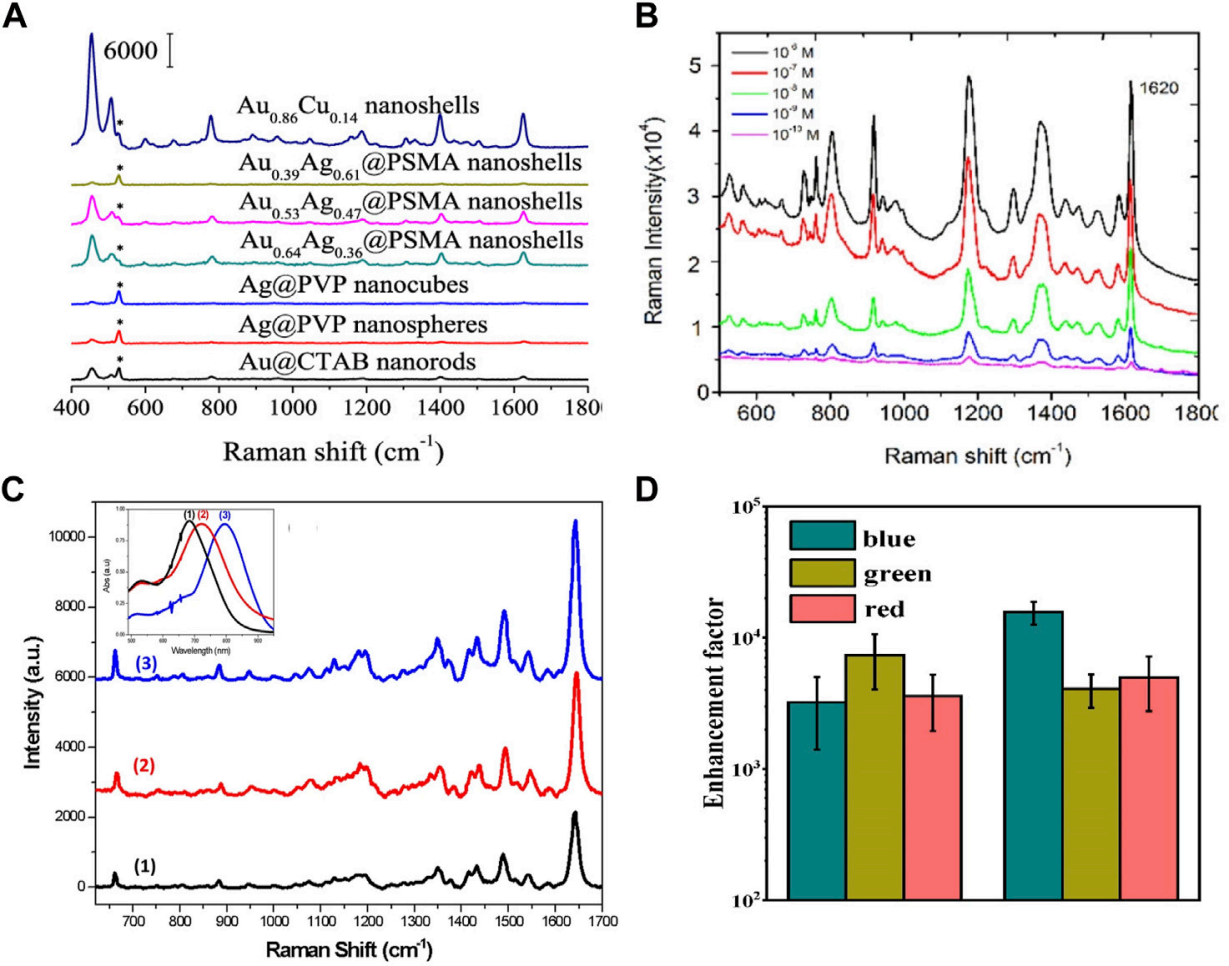
**(A)** SERS spectra with different shapes of nanostructures for MB (10^–4^ M) (Chuang et al., 2020). **(B)** SERS spectra for different concentrations of CV (10^−6^ to 10^−10^ M) (Kumar-Krishnan et al., 2020). **(C)** SERS spectra with different aspect ratios of Au_1_Cu_3_ NRs for NBA (10^–5^ M). Insets in **(C)** show extinction spectra of Au_1_Cu_3_ NR samples s (Van et al., 2022). **(D)** Enhancement factors of emission of Yb^3+^/Er^3+^-doped nanoparticles coupled with a Au–Cu Janus nanostructure (Mi et al., 2022).

Au–Cu nanostructures show LSPR tunability and larger enhancement of the near field, which can be applied in a variety of enhanced spectroscopies (Zhang et al., 2017; Chen et al., 2018; Cabello et al., 2019; Wang X. et al., 2020). To exploit the surface-enhanced Raman scattering (SERS) performance of Au–Cu nanoshells, MB molecules were used as probe molecules (Chuang et al., 2020). Figure 7A shows that the intensity of the SERS spectra at 1621 cm^−1^ for the Au–Cu nanoshells was enhanced by 10.3-fold, 16.7-fold, 26.78-fold, 2.54-fold, 2.71-fold and 22.58-fold relative to that for the Au NRs, Ag@PVP nanoparticles, Ag@PVP nanocubes, Au_0.64_–Ag_0.36_@PSMA nanoshells, Au_0.53_Ag_0.47_@PSMA nanoshells, and Au_0.39_Ag_0.61_@PSMA nanoshells, respectively. It can be demonstrated that Au–Cu nanoshells are effective and reliable for SERS detection, with promising potential applications in visualizing and sensing living bladder cancer cells in physiological environments. Kumar-Krishnan and coworkers evaluated the sensitivity of CV detection of SERS with Au–Cu flower-shaped nanostructures (Kumar-Krishnan et al., 2020). Figure 7B shows the SERS spectra of CV molecules with different concentrations (10^−6^ to 10^−10^ M) recorded over the particular substrate. In addition, Au–Cu NRs were designed by Nguyen and coworkers (Van et al., 2022). The plasmon wavelength of Au–Cu NRs can be tuned by changing the aspect ratio. The SERS performance of Au_1_–Cu_3_ NRs with different aspect ratios is investigated. As shown in Figure 7C, clearly, the SERS spectral intensities of NBA increase as the Au–Cu NRs become longer. The reason was explained by the enhancement of the local electromagnetic field intensity. Recently, Au–Cu nanostructures have been employed to enhance the luminescence of Yb^3+^/Er^3+^-doped nanoparticles (Mi et al., 2022). The plasmonic Au–Cu Janus nanojellyfish showed two LSPR peaks, which coupled the emission and excitation of the light wavelength of the Yb^3+^/Er^3+^-doped nanoparticles. A 5000-fold enhancement of the emission of Yb^3+^/Er^3+^-doped nanoparticles was achieved (Figure 7D). It is shown that multiple LSPR modes of Au–Cu Janus nanojellyfish can exhibit the potential to enhance the emission of upconversion nanoparticles.

To date, Au–Cu nanostructures have exhibited excellent enhancement of the near field in a variety of enhanced spectroscopies. Nevertheless, view from the current study, the application of Au–Cu nanostructures is just in the early stage compared with Au and Ag nanostructures. Focusing on the enhanced spectroscopies of Au–Cu nanostructures, further works is the construction high-performance Au–Cu nanostructures.

## 5 Conclusion

In summary, we have presented recent works on Au–Cu nanostructures. The fabrication strategies of Au–Cu nanostructures and their applications have been highlighted. Three types of Au–Cu nanostructures with different shapes and compositions are prepared by co-reductiont method, capping agent-directed method, seed-mediated growth method, and other methods. Au–Cu nanostructures have shown great potential in plasmon-enhanced spectroscopy, catalysis, and phototherapy fields. Although many developments have been made regarding Au–Cu nanostructures, many questions remain. The diversity of Au–Cu nanostructures is still limited by the structure. The technology for regulating the Au–Cu nanostructure is far from mature. The detailed growth mechanisms and properties of some Au–Cu nanostructures are unclear. Moreover, maintaining the long-term stability of Au–Cu nanostructures is challenging and needs more exploration. Although several types of Au–Cu Janus nanostructures have been reported, the uniformity of Au–Cu Janus requires significant improvement. Considering the importance of shape control in defining the properties of the nanocrystals, more unconventional shapes should be developed for the Au*–*Cu nanostructure. Doping, alloying, or integration to generate Au*–*Cu nanostructures may further improve their performance and broaden their applications. As a major challenge, the high susceptibility of Cu to oxidation greatly restricts the storage and utilization of Au–Cu nanostructures. To prevent the oxidation of the Au–Cu nanostructure, the new surface passivation technologies can be developed by a series of materials, such as graphene, polymers, SiO_2_, metal oxides and noble metals to protect the Au–Cu nanostructure. From anti-oxidation techniques, forming an alloy or a core–shell structure with robust materials by alloying and electroplating. Compared with alloyed structures, core–shell structures are considered more effective in protecting Cu atoms from oxidation because Cu atoms cannot be directly exposed on the surface in the ideal case.

In conclusion, there are numerous opportunities in the development of various Au–Cu nanostructures and in the exploration of their current applications. It is necessary to improve fabrication techniques in order to understand the mechanisms behind the growth and properties of Au–Cu nanostructures. This not only expands the types of Au–Cu materials but also promotes their current applications in nanomotors, biomedicine, sensing, and solar energy.
